# Evaluation of the anti-inflammatory, antinociceptive, and antipyretic potential of ethanolic extract of *Aristida depressa* Retz through *in vitro, in vivo*, and *in silico* models

**DOI:** 10.3389/fphar.2024.1326482

**Published:** 2024-07-10

**Authors:** Asmaa E. Sherif, Rabia Alam, Muhammad Asif, Kashif-ur-Rehman Khan, Muhammad Sajid Ur Rehman

**Affiliations:** ^1^ Department of Pharmacognosy, College of Pharmacy, Prince Sattam Bin Abdulaziz University, Al-Kharj, Saudi Arabia; ^2^ Department of Pharmacognosy, Faculty of Pharmacy, Mansoura University, Mansoura, Egypt; ^3^ Department of Pharmacology, Faculty of Pharmacy, The Islamia University of Bahawalpur, Bahawalpur, Pakistan; ^4^ Department of Pharmaceutical Chemistry, Faculty of Pharmacy, The Islamia University of Bahawalpur, Bahawalpur, Pakistan; ^5^ Department of Pharmacognosy, Faculty of Pharmacy, The Islamia University of Bahawalpur, Bahawalpur, Pakistan

**Keywords:** *Aristida depressa* Retz, anti-inflammatory, analgesic, antipyretic, *in silico* studies

## Abstract

Uncontrolled inflammation is a crucial factor in the development of many diseases. Anti-inflammatory molecules based on natural sources are being actively studied, among which *Aristida depressa* Retz (*Ar.dp*) has been traditionally used as a paste to heal inflammation. The present study aimed to evaluate the anti-inflammatory, analgesic, and antipyretic potential of an ethanolic extract of *A. depressa* through a battery of *in vivo* and *in vitro* models. The ethanolic extract of *A. depressa* was prepared by maceration and chemically characterized using high-performance liquid chromatography, which revealed the presence of quercetin, vanillic acid, chlorogenic acid, *p*-coumaric acid, *m*-coumaric acid, ferulic acid, cinnamic acid, and sinapic acid; its antioxidant capacity was then screened with the DPPH *in vitro* assay, which indicated moderate scavenging capacity. A protein denaturation assay was next performed to evaluate the *in vitro* anti-inflammatory potential of *Ar.dp*, which showed significant inhibition (44.44%) compared to the standard drug (diclofenac sodium), with 89.19% inhibition at a concentration of 1 mg/mL. The *in vivo* safety profile of *Ar.dp* was evaluated in accordance with the OECD-425 acute toxicity guidelines and found to be safe up to 5 g/kg. The *in vivo* anti-inflammatory potentials of *Ar.dp* were evaluated at three different doses (125, 250, and 500 mg/kg) in acute (carrageenan-induced edema: 84.60%, histamine-induced paw edema: 84%), sub-chronic (cotton-pellet-induced granuloma: 57.54%), and chronic (complete-Freund’s-adjuvant-induced arthritis: 82.2%) models. Our results showed that *Ar.dp* had significant (*p* < 0.05) anti-inflammatory effects over diclofenac sodium in the acute and chronic models. Histopathology studies indicated reduced infiltration of paw tissues with inflammatory cells in *Ar.dp*-treated animals. Similarly, *Ar.dp* showed significant (*p* < 0.05) analgesic (yeast-induced-pyrexia model: 23.53%) and antipyretic (acetic-acid-induced writhing model: 51%) effects in a time-dependent manner. *In silico* studies on the interactions of COX-1 and COX-2 with the eight ligands mentioned earlier confirmed the inhibition of enzymes responsible for inflammation and fever. Based on the findings of the present study, it is concluded that *Ar.dp* has anti-inflammatory, analgesic, and antipyretic properties that are likely linked to its pharmacologically active phenolic bioactive molecules.

## Introduction

Inflammation is a kind of defense mechanism of the immune system that slows infection progression and aids in efficient extermination of a wide range of foreign invasions. However, uncontrolled and uncoordinated inflammation may result in tissue injury, because of which it is viewed as a double-edged sword (Gojznikar et al., 2022). Chronic inflammation is considered as one of the major causes of diseases, including rheumatoid arthritis, diabetes mellitus, cardiovascular disorders, various cancers, and hypersensitivity reactions (Andleeb et al., 2022). Inflammation-induced problems have been thought to be responsible for about 25% of the malignancies reported globally (Mantovani, 2010). Inflammatory macrophages are also one of the main factors in the development of various types of cancers (Deryugina and Quigley, 2015).

Numerous studies have demonstrated that the primary reasons for the globally increasing morbidity rates are chronic inflammation and musculoskeletal abnormalities. Although swelling is a defense mechanism, chronic inflammation is characterized by excessive inflammatory reactions (Kalischuk and Buret, 2010). Prostaglandins (PGs) are some of the key players in inflammation and promote vasodilatation, pain, edema, and fever. In addition, oxidative-stress-associated TNF-α signaling plays a critical role in the development of chronic inflammation (El-Mowafy et al., 2010). The imbalance between pro- (increased levels) and anti-inflammatory (reduced levels) cytokines has been identified as a critical cause in the pathogenesis of arthritis (Wojdasiewicz et al., 2014). Therefore, multitarget therapies are being advocated over monotarget drugs to produce better effects in arthritis patients. Antioxidants are believed to have anti-inflammatory and cancer-fighting capabilities (Graham et al., 2013). Traditional arthritis treatments that include steroids, non-steroidal anti-inflammatory drugs (NSAIDs), and biological agents such as antagonists of TNF-α and interleukin-1 beta (IL-1β) have limited efficacies and are associated with several unpleasant side effects (Ahmed et al., 2005). Thus, numerous herbal medications derived from plant extracts are being used to treat a wide range of autoimmune conditions (Ratheesh and Helen, 2007).

Activation of transcription factors, stabilization of lysosomal membranes, inhibition of inflammatory enzymes (cyclooxygenase and lipoxygenase), suppression of proinflammatory cytokines release, and inhibition of arachidonic acid breakdown into inflammatory products are a few of the proposed mechanisms by which natural products are known to prevent the development of edema (Nworu and Akah, 2015). In addition, numerous clinical studies have reported the immune-suppressing and anti-inflammatory effects of plant extracts, dietary supplements, and minerals with highly encouraging results. However, there is still a lack of understanding about the action mechanisms, safety profiles, and interactions between plant extracts and functional foods or commercially available medications, which has necessitated further research in this field.

Chronic inflammatory conditions, such as arthritis, fever, and pain, impose significant economic burdens on healthcare systems owing to the expenses associated with treatment, medication, and hospitalization (Cao et al., 2016; Wang et al., 2022; Xiang et al., 2023). Evaluating the anti-inflammatory, antinociceptive, and antipyretic capabilities of natural products, such as the ethanolic extract of *Aristida depressa* Retz (Clayton et al., 2006), could potentially offer cost-effective alternatives to conventional pharmaceuticals, thereby reducing the healthcare costs for both individuals and healthcare systems (Xiong et al., 2019; Feng et al., 2023; Lin et al., 2023). Access to affordable and effective healthcare is a significant socioeconomic issue globally. Research into natural remedies like the *A. depressa* extract could enhance access to treatments for individuals in regions where conventional pharmaceuticals are either expensive or inaccessible (He et al., 2021; Liu et al., 2022a; Jiang et al., 2022; Yu et al., 2022). This could particularly benefit economically disadvantaged communities and regions with limited healthcare infrastructure. Research into natural alternatives like the extract of *A. depressa* could also impact the pharmaceutical industry through potential development of new drugs or supplements derived from natural sources (Wei et al., 2018; Hu et al., 2022; Luo et al., 2022; Yi et al., 2022; Imashiro et al., 2023); this could create economic opportunities for pharmaceutical companies involved in the research, development, and commercialization of such products (Liu et al., 2022b; Jiang et al., 2023).

*A. depressa* Retz, often known as six-weeks three-awn grass, is a species with global distribution. The presence of three awns (bristles) on the lemma of each floret distinguishes *A. depressa* from other species (Manimaran et al., 2020). *A. depressa* methanol extracts are known to have considerable antioxidant and antibacterial capabilities, which have been linked to their therapeutic properties (Amjad, 2015; Fatima et al., 2019). The whole plant parts of *A. depressa* have been used as pastes topically over damaged areas to cure wounds (Ayyanar and Ignacimuthu, 2009). *A. depressa* has also been used to treat numerous skin diseases, including inflammation (Majeed et al., 2020). However, to the best of our knowledge, there are no detailed *in vivo* studies regarding the therapeutic effects of *A. depressa* on chronic inflammation and its major molecular interactions. Therefore, the present study aimed to explore the anti-inflammatory attributes of the methanolic extract of *A. depressa* through a series of *in vivo*, *in vitro*, and *in silico* models.

## Materials and methods

### Collection and extraction of the plant material

The *A. depressa* plant was collected from the garden of Lal Sohanra National Park, Bahawalpur, Pakistan, in November 2020. The plant sample was identified by Mr. Abdul Hameed (district forest officer) and voucher no. 177 was allotted to the plant sample submitted to the Department of Agriculture and Forestry, Bahawalpur. The plants were completely dried in shade and ground coarsely. The extraction procedure involved simple maceration (shaking after 24 h at 25°C), followed by filtering with Whatman grade-1 filter paper and concentration to dryness under a reduced pressure of −760 mmHg at a temperature below I40°C using a rotary evaporator. A dark-green thick mass was obtained, which was placed in an oven for complete drying. The extract was then stored in a glass bottle and labeled as *Ar.dp.*

### Qualitative phytochemical screening

An extant method was used for qualitative analysis of *Ar.dp* to screen the different primary and secondary metabolites (Saleem et al., 2021).

### Chemical characterization by high-performance liquid chromatography (HPLC)

Gradient HPLC was performed to identify the various bioactive phytochemicals present in *Ar.dp*; the metabolites were separated using a Shimpack HPLC system (Shimadzu, Japan) fitted with a CLC-ODS (C-18) column of dimensions 25 cm × 4.6 mm and diameter 5 µm. The biphasic mobile phase used in the current study was prepared from two different compositions: mobile phase I comprised water and acetic acid in a ratio of 94:6 with a pH of 2.27; mobile phase II contained acetonitrile (ACN) run at 15% for 0–15 min, followed by 45% for 15–30 min and 100% for 30–45 min. Then, the metabolites were detected using an ultraviolet detector (280 nm wavelength), and a chromatogram was drawn between the voltage and time. The retention times of the detected metabolites were compared with those of the standard metabolites, as reported in our previous study (Asif et al., 2016a).

### Antioxidant assay

Six different concentrations of *Ar.dp* (6.25–200 μg/mL) and ascorbic acid (standard) were prepared. About 100 μL of each dilution of the sample was added along with 100 µL of DPPH reagent to each well of a 96-well plate and incubated in the dark for 30 min. Then, the absorbances were measured at 517 nm, and the IC_50_ values were calculated using linear regression (R^2^) equations. The percentage radical scavenging activity from three independent experiments (*n* = 3) was presented in terms of the mean ± SD (Saleem et al., 2021).

### *In vitro* protein denaturation assay

The reaction mixture (5 mL) contained 0.2 mL of 1% bovine serum albumin, 4.78 mL of phosphate-buffered saline (PBS, pH = 6.4), and 0.02 mL of *Ar.dp* that was mixed and incubated in a water bath (37°C) for 15 min before being heated at 70°C for 5 min. After cooling, the turbidity was measured at 660 nm using a UV/VIS spectrophotometer, and the percentage inhibition of precipitation was calculated by the following equation (Asif et al., 2020b):

$$Percentage\;inhibition=\mathrm{below mentioned formula}=\frac{Absorbance\;of\;control-Absorbance\;of\;sample\;}{Absorbance\;of\;control\;}\times 100$$

### *In vivo* experiments

Rats (male/female) were housed in an animal facility at Islamia University of Bahawalpur under a controlled environment with 50% humidity, 25°C temperature, and 12-h light/dark cycles. Before the *in vivo* activity measurements, the animals were acclimatized for 1 week as well as being provided pellet meal and water continuously. All animal handling methods in this study were authorized by the animal ethics committee of Islamia University Bahawalpur, Pakistan (AEC file no. PAEC/21/49).

### Experimental design

To assess the anti-inflammatory properties of *Ar.dp*, albino rats were divided into five groups (*n* = 6 each). Group 1 received distilled water (10 mL/kg) orally as a negative (disease) control. The animals in Groups 2, 3, and 4 respectively received 125, 250, and 500 mg/kg of *Ar.dp* orally as a pretreatment. The animals in Group 5 received 20 mg/kg diclofenac sodium as a positive control.

### Carrageenan- and histamine-induced paw edema models

Each animal was injected with 0.1 mL of freshly prepared 1% (w/v) carrageenan and histamine solutions in the subplantar region of the right hind paw after 1 h of pretreatment (Saadullah et al., 2022). The paw volumes were measured using a digital vernier caliper at 0, 1, 2, 3, 4, 5, and 6 h intervals, and the percentage of paw edema inhibition was estimated using the following formula:

$$Percentage\;inhibition=\frac{Absorbance\;of\;control-Absorbance\;of\;sample\;}{Absorbance\;of\;control\;}\times 100$$

### Cotton-pellet-induced granuloma model

The rats were sedated with a subcutaneous injection of ketamine + xylazine mixture (1 mL/animal). A pouch was made in the groin region of each rat using sharp scissors and blunt forceps for the insertion of an autoclaved cotton ball (30 mg) subcutaneously. The rats were given appropriate doses of the plant extract and standard 4–5 h after implantation, and this dosing was continued for 14 days. Chloroform was used to anesthetize the rats on day 15. For hematological and biochemical investigations, blood was drawn by the heart puncture technique. The cotton pellets were retrieved from the rats before being dried and weighed. The percentage inhibition of granuloma formation was then expressed as mean ± SD (Sultana et al., 2019).

### Chronic inflammatory model: complete Freund’s adjuvant (CFA)-induced arthritis model

After administering *Ar.dp*, 0.1 mL of 0.4% CFA was injected intradermally into each animal’s paw. The extract administration was continued daily for the next 21 days. On days 1, 3, 5, 9, 13, and 21, each rat’s paw volume and bodyweight were measured, and the percentage inhibition of arthritis was computed. The primary and secondary lesions were among the arthritic parameters used for assessment. The development of edema in the injected paws after 3–5 days following phlogistic challenge was referred to as a primary lesion and noted on the fifth day. Secondary lesions included inflammation at non-injected sites and reduced bodyweight after 11–12 days owing to immunological responses (Nagarkar and Jagtap, 2017).

### Analgesic activity: acetic-acid-induced writhing model

The acetic-acid-induced writhing model was adopted in accordance with the method of Sajid-Ur-Rehman et al. (2021) with slight modifications. The rats (220–280 g) in this experiment were divided into four groups of six animals each. Then, the analgesic effects of an aqueous ethanolic extract of *A. depressa* against acetic-acid-induced writhing (0.6% v/v in saline, intraperitoneal) were evaluated at doses of 125, 250, and 500 mg/kg orally. The number of writhing movements (contraction/extension of the abdominal muscles) displayed by each animal was tallied for 10 min, starting from 5 min after injection of acetic acid, and the percentage inhibition was calculated by the following formula (Saravanan et al., 2014):

$$Percentage\;inhibition=\frac{No.\;of\;writhings\;\left(control\right)-No.\;of\;writhings\;\left(sample\right)\;}{No.\;of\;writhings\;\left(control\right)\;}\times 100$$

### Antipyretic activity: yeast-induced pyrexia in rats

Five groups of rats were assessed in this experiment (*n* = 5). The animals were fasted for 24 h before the trials and were denied water during the experiments. A digital thermometer was used to record the initial rectal temperature. Pyrexia was induced by injecting a 20% (w/v) brewer’s yeast suspension (10 mL/kg) subcutaneously into the tail vein. Only those rats that showed a temperature increase of at least 0.7°C were employed in the subsequent experiments when their temperatures were at the peak values 18 h after yeast injection. Group I (control group) was treated with distilled water (10 mL/kg orally); groups II, III, and IV received oral doses of *Ar.dp* at 125, 250, and 500 mg/kg, respectively; group V was treated with standard diclofenac sodium at 20 mg/kg orally. The rectal temperatures of the animals were measured at 0, 1, 2, 3, and 4 h (Metowogo et al., 2008).

### *In silico* studies

#### Preparation of enzyme and ligand molecules

Molecular docking analysis was carried out using different tools, such as Discovery Studio, Babel, AutoDock Vina, PyRx, and MGL Tools (Shahid et al., 2023). The ligand structures (3D) were downloaded from PubChem database in structure-data file (SDF) format and optimized using Babel. High-resolution COX-1 and COX-2 enzymes (Borbulevych et al., 2004) were downloaded from the RSCB Protein Data Bank (PDB; accessed on 15 May 2023) and were prepared using Discovery Studio 2021 Client by removing all inhibitors and water molecules before docking.

#### Docking interactions of the phytochemicals

The ligands and prepared enzymes were uploaded to Vina, embedded in PyRx, and docked (Khan et al., 2023). This is a useful tool in computer-aided drug development studies. First, the protein molecules (COX-1 and COX-2) were retrieved in PDB format; the PDB IDs of COX-1 and COX-2 were 3n8w and 5ikq, respectively. Then, these proteins were prepared using Discovery Studio 2021 Client. All chains, except for the A chain and active sites with already attached water molecules and ligands, were then eliminated from the protein molecules. Subsequently, polar hydrogen molecules were introduced to the proteins, and the resulting structures were saved as PDB files. Secondary metabolites chosen from the HPLC analytical technique table and standard metabolites were downloaded from the PubChem database in SDF format. Then, the prepared protein molecules were uploaded to PyRx software and subjected to the autodocking and macromolecule options. The ligands were then uploaded to PyRx from Open Babel to undergo the necessary preparation steps. Thereafter, they were minimized and converted to the PDBQT format. The grid boxes were next formed with specific dimensions for COX-1 (X: 75.0935, Y: 59.5137, Z: 61.6412) and COX-2 (X: 77.0125, Y: 62.5143, Z: 57.5410). Finally, the interactions were visualized using Discovery Studio. The docking procedures were validated by redocking the cocrystalized ligands with these enzymes.

#### Statistical analysis

The results were expressed in terms of mean ± standard error of the mean (SEM) (*n* = 6). Data among the groups were compared using two-way ANOVA, followed by Bonferroni’s test, using GraphPad Prism (San Diego, CA, United States) software. A *p-*value <0.05 was considered to be statistically significant in all cases.

## Results

### Qualitative phytochemical analysis

Data from the preliminary phytochemical analysis of *Ar.dp* indicated the presence of different classes of metabolites, including flavonoids, glycosides, alkaloids, phenols, and tannins.

### HPLC analysis

**FIGURE 1:**
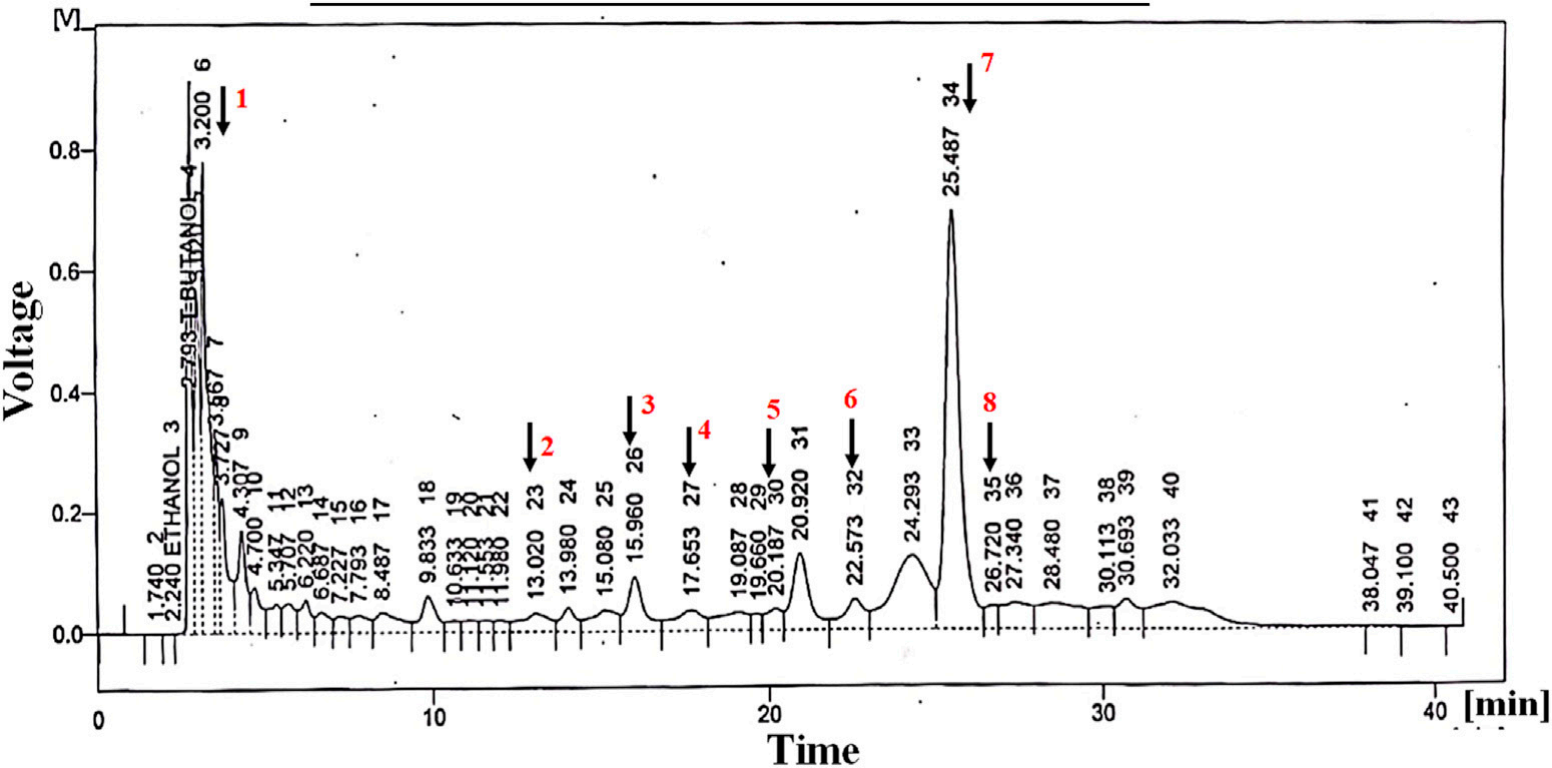
Metabolites identified by HPLC analysis of *Ar.dp.* These were verified by comparing the retention times of the peaks with standard metabolites.

HPLC analysis of *Ar.dp* indicated the presence of various phenols and flavonoids, including quercetin, vanillic acid, chlorogenic acid, *p*-coumaric acid, *m*-coumaric acid, ferulic acid, cinnamic acid, and sinapic acid (Figure 1; Table 1).

**TABLE 1:** Metabolites identified in *Ar.dp* through HPLC.

Peak	Metabolites	Retention time	Area %
1	Quercetin	3.20	9.3
2	Vanillic acid	13.02	1.7
3	Chlorogenic acid	15.96	2.8
4	*p*-coumaric acid	17.63	1.9
5	*m*-coumaric acid	20.18	1.1
6	Ferulic acid	22.57	2.1
7	Cinnamic acid	25.48	19.8
8	Sinapic acid	26.7	0.9

### *In vitro* antioxidant activities of *Ar.dp*

The IC_50_ values of *Ar.dp* and ascorbic acid were found to be 70.9 ± 0.39 and 85.51 ± 0.38 μg/mL, respectively, as shown in Table 2.

**TABLE 2:** Antioxidant activity of *Ar.dp*.

Peak	*Ar.dp*	Ascorbic acid
IC_50_ values	70.9 ± 0.39 µg/mL	85.51 ± 0.38 µg/mL

### Protein denaturation assay

**FIGURE 2:**
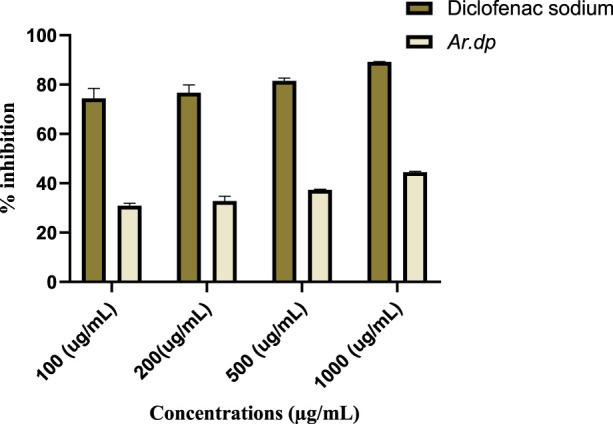
Effects of *Ar.dp* and diclofenac sodium on *in vitro* protein denaturation.

*Ar.dp* and diclofenac sodium inhibited 89.19% and 44.44% of protein denaturation at concentrations of 1 mg/mL, respectively, and their dose-dependent effects were observed for protein precipitations at 1000, 500, 200, and 100 mg/mL (Figure 2).

### Evaluation of anti-inflammatory effects of *Ar.dp* in carrageenan- and histamine-induced acute edema models

*Ar.dp* treatment exhibited dose-dependent decreases in inflammation in both of the acute inflammatory models (carrageenan- and histamine-induced edema). The *Ar.dp* treatment groups showed almost similar anti-inflammatory effects up to 4 h with no statistical differences (*p* > 0.05) in the carrageenan-induced paw edema model. From 5 h onward, *Ar.dp* at 500 mg/kg showed significantly better (*p* < 0.05) anti-inflammatory effects compared to the lower doses. Diclofenac sodium (20 mg/kg) showed significantly better (*p* < 0.05) effects compared to 500 mg/kg of *Ar.dp*. The maximum anti-inflammatory effects in terms of the percentage inhibitions of paw edema in the animals treated with 125, 250, and 500 mg/kg of *Ar.dp* and 20 mg/kg of diclofenac sodium were found to be 56.45 ± 17.34, 81.38 ± 7.7, 84.60 ± 3.4, and 84.56 ± 0.5, respectively (Table 4).

Data from the histamine-induced paw edema model showed time-dependent anti-inflammatory effects in the *Ar.dp* treatment groups. No statistical differences (*p* > 0.05) were observed in terms of the percentage inhibition of paw edema up to 3 h in both the *Ar.dp* and diclofenac sodium treatment groups. At 4 h post histamine administration, *Ar.dp* at 250 mg/kg showed better (*p* < 0.05) effects than at 125 mg/kg, and 500 mg/kg of *Ar.dp* showed better (*p* < 0.05) effects than 250 mg/kg of *Ar.dp*. When the percentage inhibition of paw edema in the 500 mg/kg of *Ar.dp* group was compared with the diclofenac sodium (20 mg/kg) treatment group, the latter showed significantly less (*p* < 0.05) inflammation. The maximum anti-inflammatory effects in terms of the percentage inhibitions of paw edema in the animals treated with 125, 250, and 500 mg/kg of *Ar.dp* and 20 mg/kg of diclofenac sodium are shown in Table 3.

**TABLE 3:** Evaluation of the anti-inflammatory effects of *Ar.dp* in the carrageenan- and histamine-induced paw edema models.

Carrageenan-induced paw edema					Histamine-induced paw edema			
Time (h)	Diclofenac 20 mg/kg	*Ar.dp* 125 mg/kg	*Ar.dp* 250 mg/kg	*Ar.dp* 500 mg/kg	Diclofenac 20 mg/kg	*Ar.dp* 125 mg/kg	*Ar.dp* 250 mg/kg	*Ar.dp* 500 mg/kg
1	25.12 ± 6.3	7.87 ± 6.2	18.92 ± 11.4	31.97 ± 10.9	37.93 ± 2.6	13.67* ± 4.9	21.48 ± 1.8	31.97* ± 0.91
2	35.97 ± 4.1	16.94 ± 4.9	30.58 ± 11.1	41.09 ± 0.4	48.95 ± 2.5	23.18* ± 8.01	29.94 ± 3.9	41.09* ± 2.4
3	47.37 ± 1.4	20.52* ± 11.1	42.28 ± 8.1	51.28* ± 5.7	54.95 ± 5.8	24.7** ± 1.9	38.99 ± 3.2	51.28** ± 0.7
4	65.81 ± 5.1	35.81 ± 13.47	59.43 ± 13.1	74.34 ± 3.8	80.18 ± 0.4	26.30 ± 9.1	49.71 ± 7.7	74.34** ± 7.2
5	74.23 ± 1.2	45.78* ± 14.65	73.83 ± 13.7	81.48* ± 4.4	89.25 ± 1.4	27.38 ± 13.39	54.64 ± 6.0	81.48 ± 0.3
6	84.56 ± 0.5	56.45* ± 17.34	81.38 ± 7.7	84.60* ± 3.4	90.53 ± 1.3	30.80 ± 17.11	55.05 ± 2.5	84.60* ±2.2

Values are expressed as Mean ± SD of the percentage inhibition of paw edema (*n* = 6); * = *p <* 0.05, ** = *p <* 0.01, and *** = *p <* 0.001.

### Anti-inflammatory effects of *Ar.dp* in the cotton-pellet-induced granuloma model

*Ar.dp* (125, 250, and 500 mg/kg) and diclofenac sodium (20 mg/kg) reduced cotton-pellet-induced granuloma formation by 35.04%, 57.54%, 43.01%, and 68.46%, respectively. Here, *Ar.dp* showed significantly (*p* < 0.05) suppressed granuloma development in a dose-dependent manner, and its effects were almost equivalent (*p* < 0.05) to those of diclofenac sodium (20 mg/kg) (Table 4).

**TABLE 4:** Anti-inflammatory effects of *Ar.dp* in the cotton-pellet-induced granuloma model.

Treatment groups	Inhibition (%)
***Ar.dp*** (125 mg/kg)	35.04 ± 4.88
***Ar.dp*** (500 mg/kg)	43.01 ± 4.14*
***Ar.dp*** (250 mg/kg)	57.54 ± 2.93*
**Diclofenac** (20 mg/kg)	68.46 ± 2.75***/ns

Values are shown as Mean ± SD of the percentage inhibitions of paw edema (n = 6); ns = non-significant, * = *p <* 0.05, ** = *p <* 0.01, and *** = *p <* 0.001.

### Anti-inflammatory effects of *Ar.dp* in the CFA-induced arthritis model

One day after CFA administration, the percentage reductions of paw edema in the groups treated with 125, 250, and 500 mg/kg *Ar.dp* and 20 mg/kg diclofenac sodium were 15.66, 21.33, 33.01, and 38.53, respectively. After the 21st day of treatment, the percentage inhibitions of paw edema were 30.06, 34.39, 82.2, and 89.89 in the groups treated with 125, 250, and 500 mg/kg of *Ar.dp* and 20 mg/kg of diclofenac sodium, respectively (Table 5). A comparison between the percent reductions of paw edema in the groups treated with *Ar.dp* at 500 mg/kg and 20 mg/kg of diclofenac sodium showed non-significant difference (*p* > 0.05).

**TABLE 5:** Anti-inflammatory effects of *Ar.dp* in the CFA-induced arthritis model (oral administration).

Time (days)	Diclofenac 20 (mg/kg)	*Ar.dp* 125 (mg/kg)	*Ar.dp 25*0 (mg/kg)	*Ar.dp* 500 (mg/kg)
1	38.53 ± 6.6	15.66 ± 6.0*	21.33 ± 7.2**	33.01 ± 0.1***
3	43.99 ± 20.8	14.39 ± 11.5*	22.08 ± 8.07**	35.23 ± 12.9***
7	46.97 ± 8.02	20.03 ± 12.6*	27.7 ± 5.6**	44.96 ± 10.8***
9	60.53 ± 18.27	23.03 ± 12.7*	26.88 ± 11.09**	55.31 ± 12.4***
13	74.54 ± 15.39	25.12 ± 11.75*	28.91 ± 7.33**	63.16 ± 5.2***
21	89.89 ± 7.4	30.06 ± 13.26*	34.39 ± 10.7**	82.2 ± 8.5***

Values are expressed as Mean ± SD of the percentage inhibition of paw edema (*n* = 6); * = *p <* 0.05, ** = *p <* 0.01, and *** = *p <* 0.001.

### Histopathological evaluation of paw samples in the CFA-induced arthritis model

**FIGURE 3:**
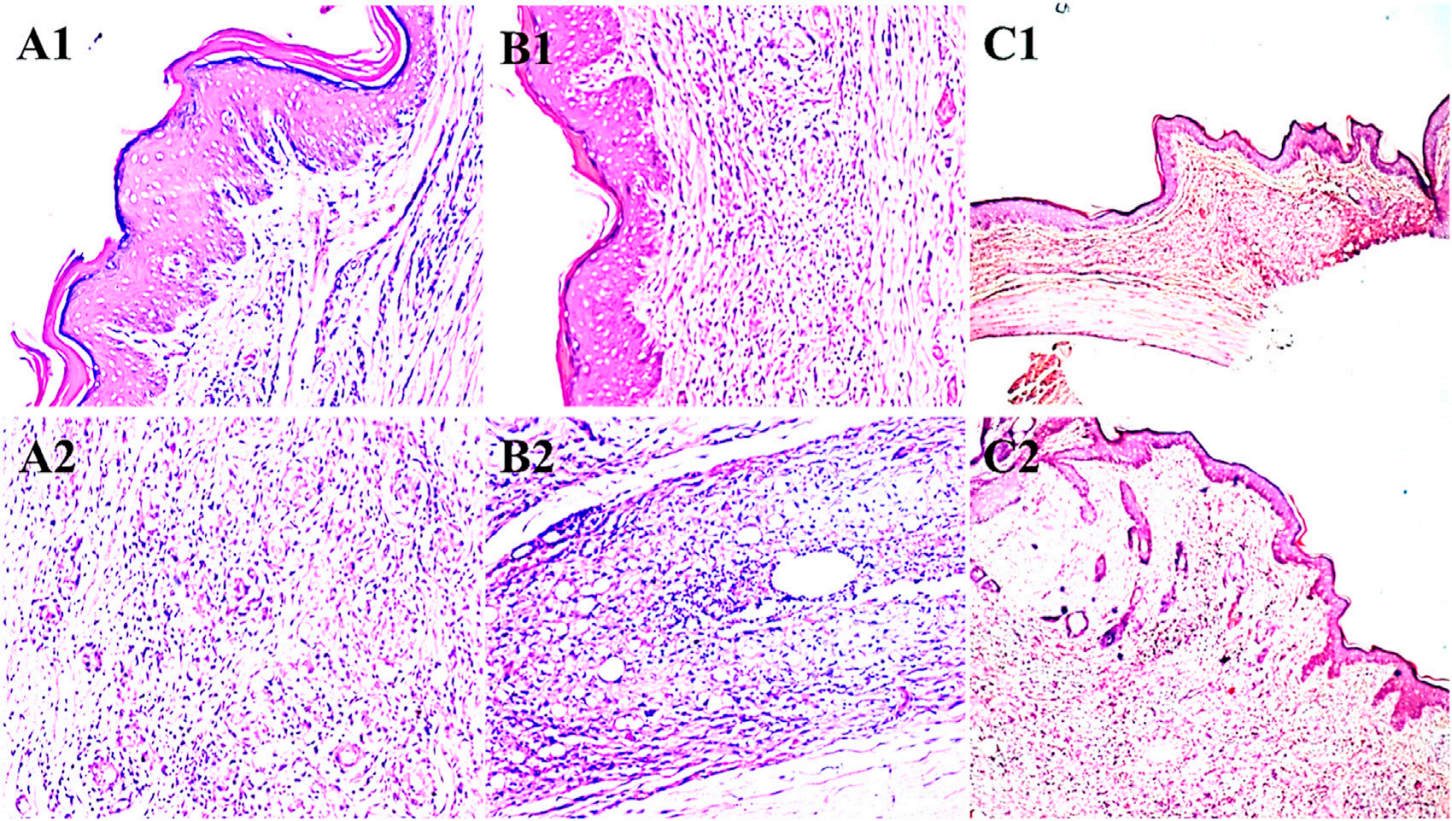
Histopathological changes observed in the paws of animals treated with **(A1, A2)** diclofenac sodium, **(B1, B2)** disease control, and **(C1, C2)** 500 mg/kg of *Ar.dp*.

Microscopic examinations of the paws in the diclofenac sodium (20 mg/kg) treatment group showed epithelial hyperplasia and inflammation in the wounded areas of the paws, with lymphocyte infiltration and granuloma formation (A1 and A2). The paws of the rats in the disease control group showed epithelial hyperplasia, hyperkeratosis, and inflammation in the wounded areas along with capillary proliferation and lymphocyte formation. The paws of the animals treated with 500 mg/kg of *Ar.dp* showed mild epithelial hyperplasia, hyperkeratosis, and reduced inflammation in the wounded areas (Figure 3).

### Analgesic effects of *Ar.dp* in the acetic-acid-induced writhing model

The analgesic effects of different treatments were calculated through the reductions in the number of writhing movements caused by administration of acetic acid. The percentage inhibitions of aberrant writhings were calculated to be 21, 31, 51, and 57 in the groups treated with 125, 250, and 500 mg/kg of *Ar.dp* and 20 mg/kg of diclofenac sodium, respectively. There was considerable analgesic activity at 500 mg/kg when compared to the animal groups treated with 125 and 250 mg/kg of *Ar.dp* (*p* ≤ 0.05 and *p* ≤ 0.01). The percentage suppression of aberrant writhings was not significantly different between the groups treated with *Ar.dp* at 500 mg/kg and diclofenac at 20 mg/kg (Table 6).

**TABLE 6:** Analgesic effects of *Ar.dp* in the acetic-acid-induced model.

Time (min)	Diclofenac 20 (mg/kg)	*Ar.dp* 125 (mg/kg)	*Ar.dp 25*0 (mg/kg)	*Ar.dp* 500 (mg/kg)
15–20	57 ± 0.9	21 ± 6.68*	31 ± 4.7**	51 ± 2.6***

Values are expressed as Mean ± SD of the percentage inhibition of analgesic activity (*n* = 6); * = I 0.05, ** = *p <* 0.01, and *** = *p <* 0.001.

### Antipyretic effects of *Ar.dp* in yeast-induced hyperthermia model

The antipyretic activity was evaluated through fever induced by injection of Brewer’s yeast. After 18 h, the animals were given doses of 125, 250, and 500 mg/kg of *Ar.dp*. The percentage inhibitions of fever in the groups treated with 125, 250, and 500 mg/kg of *Ar.dp* vs. 20 mg/kg of diclofenac were 9.15, 11.30, 23.53, and 21.03 over the first hour after administration as well as 35.16, 39.08, 71.78, and 85.45 in the fourth hour after administration, respectively. There was significant activity in terms of the percentage inhibition of fever when comparing the animals treated with *Ar.dp* at 500 mg/kg to those administered diclofenac sodium at 20 mg/kg (*p* < 0.05) as shown in Table 7.

**TABLE 7:** Antipyretic effects of *Ar.dp* in the yeast-induced hyperthermia model.

Time (days)	Diclofenac 20 (mg/kg)	*Ar.dp* 125 (mg/kg)	*Ar.dp 25*0 (mg/kg)	*Ar.dp* 500 (mg/kg)
1	21.03 ± 6.1	9.15 ± 2.1*	11.30 ± 4.2**	21.03 ± 4.2***
3	37.91 ± 17.09	20.19 ± 6.8*	23.11 ± 5.1**	37.91 ± 0.6***
7	60.41 ± 2.9	29.53 ± 3.69*	33.13 ± 3.6**	60.41 ± 3.4***
10	85.41 ± 2.9	35.16 ± 0.7*	39.08 ± 0.2**	85.41 ± 4.5***

Values are expressed as Mean ± SD of the percentage inhibition of antipyretic activity (*n* = 6); * = *p* < 0.05, ** = *p* < 0.01, and *** = *p* < 0.001.

### *In silico* studies: molecular docking of metabolites against COX-1 and COX-2

**FIGURE 4:**
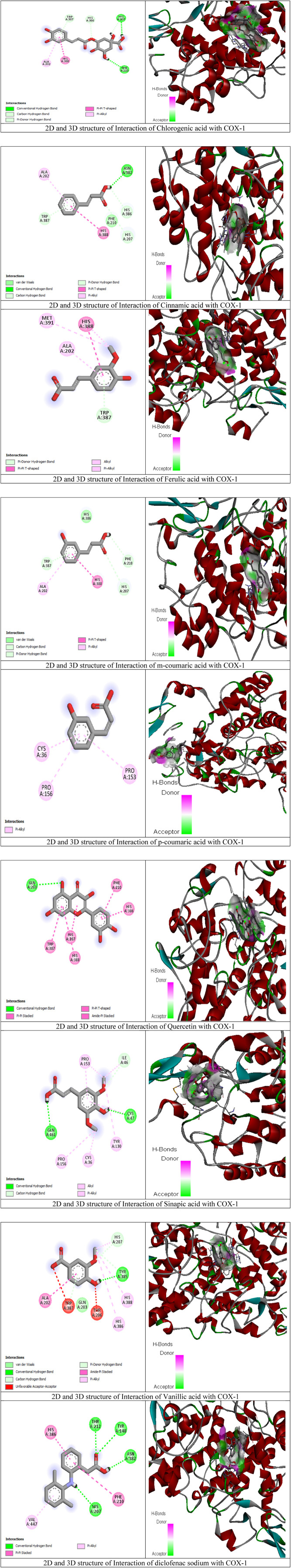
Molecular docking results of chlorogenic acid, cinnamic acid, ferulic acid, *m*-coumaric acid, *p*-coumaric acid, quercetin, sinapic acid, vanillic acid, and diclofenac sodium with COX-1 proteins.

**FIGURE 5:**
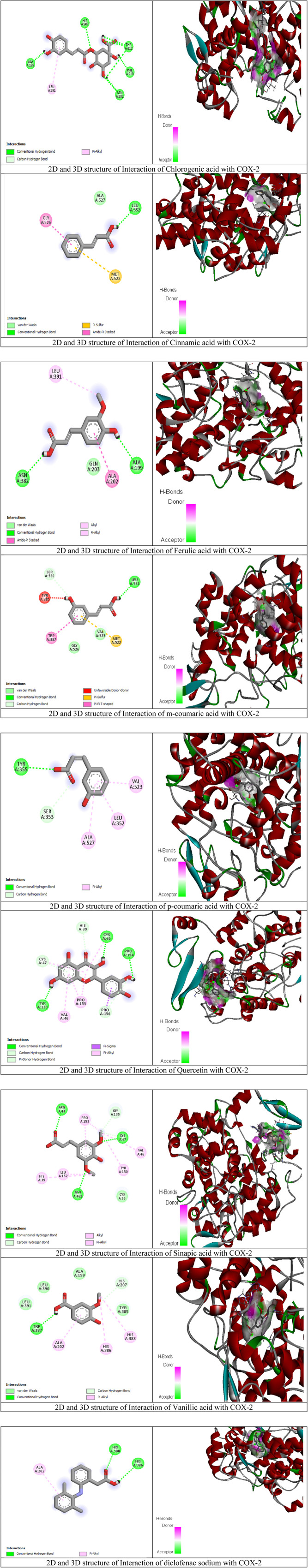
Molecular docking results of chlorogenic acid, cinnamic acid, ferulic acid, m-coumaric acid, p-coumaric acid, quercetin, sinapic acid, vanillic acid, and diclofenac sodium with COX-2 proteins.

To better assess the inhibition potentials of the metabolites and to compare their data with enzyme inhibition findings, the eight metabolites identified by HPLC and one standard compound were docked against the COX-1 and COX-2 proteins. Quercetin showed the highest binding affinity (−9.7) among all phytometabolites reported by HPLC, while diclofenac sodium (standard, −6.5) presented the maximum binding affinity with the COX-1 proteins (Table 8, Figure 4). Similarly, for the COX-2 proteins, quercetin showed the highest binding affinity (−8.3), while diclofenac sodium (standard, −6.7) presented the maximum binding affinity (Table 9, Figure 5).

**TABLE 8:** Binding affinities of all reported phenolic metabolites and standard drugs with COX-1 proteins.

Metabolites	Binding affinity
Chlorogenic acid	−8.1
Cinnamic acid	−6.2
Ferulic acid	−6.7
m-coumaric acid	−6.6
p-coumaric acid	−5.1
Quercetin	−9.7
Sinapic acid	−6.8
Vanillic acid	−6.3
Diclofenac sodium	−6.5

**TABLE 9:** Binding affinities of all reported phenolic metabolites and standard drugs with COX-2 proteins.

Compound	Binding affinity
Chlorogenic acid	−7.9
Cinnamic acid	−6.5
Ferulic acid	−6.6
m-coumaric acid	−6.5
p-coumaric acid	−6.7
Quercetin	−8.3
Sinapic acid	−6.8
Vanillic acid	−6.1
Diclofenac sodium	−6.7

## Discussion

The present study entailed estimation of the anti-inflammatory, antinociceptive, and antipyretic efficacies of an ethanolic extract of *A. depressa* Retz *in vitro* and *in vivo* in rats as well as confirmation of this potential through *in silico* studies. It has been reported that the pharmacological effects of plants are typically exerted by the presence of antioxidant substances like phenols, phenolic diterpenes, and flavonoids. Our data from the preliminary phytochemical analysis of *A. depressa* suggest the presence of flavonoids, glycosides, alkaloids, phenols, and tannins.

Phenolic metabolites are a class of naturally occurring substances that are primarily composed of phenolic acids and flavonoids; they are found throughout the plant kingdom. Phenols and flavonoids are known to have broad-spectrum free-radical scavenging properties (Asif et al., 2016b). The HPLC analysis in this study estimated the polyphenolic content of *Ar.dp* and showed the presence of various phenolic and flavonoid components. Data from the DPPH free-radical assay also indicated moderate antioxidant power of the extract. Based on the chemical composition and antioxidant potential, it is suggested that the protein denaturation prevention mechanism of *Ar.dp* is through the blockage of free-radical-mediated protein damage.

Denaturation of tissue proteins is a common occurrence in various chronic inflammatory diseases, including arthritis. Denatured proteins act as proinflammatory agents and also produce autoantigens in various inflammation-associated disorders, including rheumatoid arthritis, glomerulonephritis, serum sickness, and systemic lupus erythematosus, thus aggravating the diseased condition further through overactivation of the immune system (Osman et al., 2016). The first screening assay investigated the *in vitro* capacity of *Ar.dp* to prevent or minimize denaturation of tissue proteins using egg albumin. The obtained data indicate that *Ar.dp* inhibits denaturation of egg albumin (Griffiths et al., 1988).

Carrageenan is commonly employed to evaluate the anti-inflammatory efficacies of natural drugs; it is known to activate tissue macrophages that then produce and secrete various proinflammatory cytokines, such as bradykinin, histamine, tachykinins, TNF-α, and IL-1β. Reactive oxygen species (ROS)-mediated activation of various cell-signaling pathways is known to play a central role in the production of these proinflammatory cytokines (Asif et al., 2020a; Lopes et al., 2020). Histamine is an irritant that may increase the production of reactive oxygen and nitrogen species in the subplantar region of the hind paws, thereby resulting in edema, hyperalgesia, and erythema at the site of inflammation (Amdekar et al., 2012; Yang et al., 2021). Cellular infiltration (neutrophils) also plays a major role in inflammation. Our findings indicated that *Ar.dp* produces a marked reduction in the cellular infiltration of neutrophils and leukocytes in the carrageenan-treated rat paws. Treatment with an ethanolic extract of *Ar.dp* inhibited the development of paw edema in both the carrageenan- and histamine-induced acute inflammation models in the present study.

Cotton-pellet-induced granuloma is a proliferative stage of inflammation that involves migration of white blood cells (WBCs). It is a common method for the development of chronic inflammation and mainly involves monocyte infiltration, fibroblast proliferation, angiogenesis, and exudation (Meshram et al., 2016). *Ar.dp* demonstrated substantial inhibitory effects in cotton-pellet-induced inflammation, implying that it can inhibit inflammatory mediator release and their harmful effects in the experimental animals. These findings show the effectiveness of *Ar.dp* in suppressing fibroblasts as well as the synthesis of collagen and mucopolysaccharides, thereby making it a natural potent supplement capable of inhibiting granuloma formation and suppressing the proliferative phase of the inflammation process.

The CFA-induced model is a well-characterized method for targeting inflammatory arthritic pain. After 3 days, neutrophils and myeloperoxidase (MPO)-positive cells flooded the inflammatory core; by day 5, the monocytes and macrophages as well as macrophage-specific proteins (Iba1) from the periphery had invaded this core, and the ailments were accompanied by substantial oxidative stress (Asgar et al., 2015). CFA-induced inflammatory cell infiltration was significantly decreased by *Ar.dp*, suggesting its promising effects on chronic inflammation and arthritis. In addition, *Ar.dp* showed a binding affinity with the sigma-2 receptors that have recently been shown to be involved in neuropathic pain.

*Ar.dp* also showed analgesic and antipyretic effects by impeding the release of central and peripheral inflammatory mediators. Pain is interlinked with oxidative stress; therefore, the presence of antioxidant metabolites in *Ar.dp* may also be responsible for its analgesic properties. HPLC analysis confirmed sufficient quantities of phenols and phenolic acids in the methanolic extract of *A. depressa*; quercetin possesses free-radical scavenging capability, while vanillic acid, *m*-coumaric acid, and *p*-coumaric acid have anti-inflammatory, analgesic, and antioxidant properties (Alamgeer et al., 2016). Therefore, in the present study, the capability of *Ar.dp* in lowering the synthesis of endogenous inflammatory mediators supports its historic use in the treatment of inflammatory disorders, pain, and pyrexia.

Molecular docking was employed to theoretically assess the interactions between the ligands and enzymes, with the aim of comprehending the molecular mechanisms responsible for the diverse biological activities exhibited by natural products against inflammation, analgesia, and fever. To understand the inhibition potentials of the studied metabolites and to compare the enzyme inhibitions of inflammation, eight metabolites found by HPLC (chlorogenic acid, cinnamic acid, ferulic acid, *m*-coumaric acid, *p*-coumaric acid, quercetin, sinapic acid, and vanillic acid) as well as diclofenac sodium (standard) were docked against COX-1 and COX-2. Conclusively, the molecular docking results show the interactions of COX-1 and COX-2 with the eight ligands, confirming our findings for the plant extract in terms of the COX-1 and COX-2 inhibition assays.

## Conclusion

The current work demonstrates that an ethanolic extract of *A. depressa* exhibits remarkable anti-inflammatory, antinociceptive, and antipyretic properties based on *in vivo* and *in vitro* assays; therefore, this extract has potential to be used in the treatment of numerous inflammatory diseases. The presence of phenolic acids, flavonoids, and antioxidants in the ethanolic extract is considered to be responsible for its anti-inflammatory, antipyretic, and analgesic properties. *In silico* analysis of the inflammatory proteins COX-1 and COX-2 enzymes against the bioactive metabolites of *Ar.dp* show high binding affinities with both proteins, thereby validating the anti-inflammatory potential of *A. depressa.* Finally, we propose that further exploration of *A. depressa* will enable its use as a new potential therapeutic agent for clinical treatment of inflammation and related disorders in the future.

## Data Availability

The original contributions presented in the study are included in the article/supplementary material, and any further inquiries may be directed to the corresponding author.
